# *Nocardia brasiliensis* Induces Formation of Foamy Macrophages and Dendritic Cells *In Vitro* and *In Vivo*

**DOI:** 10.1371/journal.pone.0100064

**Published:** 2014-06-17

**Authors:** Irene Meester, Adrian Geovanni Rosas-Taraco, Mario Cesar Salinas-Carmona

**Affiliations:** Department of Immunology, Faculty of Medicine, Universidad Autónoma de Nuevo León, Nuevo León, México; Duke University Medical Center, United States of America

## Abstract

Foamy cells have been described in various infectious diseases, for example in actinomycetoma induced by *Nocardia brasiliensis*. These cells are generally considered to be macrophages, although they present dendritic cell (DC)-specific surface markers. In this study, we determined and confirmed the lineage of possible precursors of foamy cells *in vitro* and *in vivo* using an experimental actinomycetoma model in BALB/c mice. Bone marrow-derived macrophages (BMDM) or DC (BMDC) were infected *in vitro* with *N. brasiliensis* or labeled with carboxyfluorescein diacetate succinimidyl ester (CFSE). Both, macrophages and DC, differentiated into foamy cells after *in vitro* infection. CFSE-labeled BMDM or BMDC were tested for phagocytosis and CD11c/CD11b receptors markers expression before being transferred into the actinomycetoma lesion site of infected mice. *In vivo* studies showed that BMDM and BMDC were traced at the site where foamy cells are present in the experimental actinomycetoma. Interestingly, many of the transferred BMDM and BMDC were stained with the lipid-droplet fluorophore Nile Red. In conclusion, macrophages and DC cells can be differentiated into foamy cells *in vitro* and *in vivo* during *N. brasiliensis* infection.

## Introduction

Lipid-laden foamy cells may appear in bacterial infections some of them; produce world health problems, such as malaria and tuberculosis. In fact, members the four main groups of pathogens, virus [1], [2], bacteria [3], [4], fungi [5] and parasites [6] have been related with the presence of lipid-laden cells in infected tissues. Prostaglandins production and foamy cells may appear as part of the host response [7], [8], reports suggest that foamy cells may favor pathogen persistence in the host [9]. For example, foamy cells in leprosy are related with the aggressive lepromatous form of the disease [10], [11]. Foamy cells in tuberculosis play a role as a refuge for dormant *Mycobacterium tuberculosis* [4], which has switched to a lipid-based metabolism [12]. Biochemical analysis of the caseous material showed that its main components were triglycerides and cholesterol, suggesting that the accumulated lipids are not indigestible remnants of the micobacterial cell wall but rather host-derived lipids [13].

Foamy cells have also been observed in the actinomycetoma that may develop after accidental inoculation with the saprophyte *Nocardia brasiliensis* [14]. *N. brasiliensis* belongs to the Actinomycetes, as do *Mycobacteria* spp., and they share a similar architecture of the cell wall with abundant mycolic acids, which are important virulence factors [15], [16]. However, *N. brasiliensis* has a larger genome, which encodes more proteins that enable the bacteria to deal with the harsh, ever-changing environments of the soil or to develop resistance to antibiotics [17]. Actinomycetoma by *N. brasiliensis* is endemic in México, and failure of antibiotic treatment due to resistance development often leads to amputation as a final option of treatment [18].

In order to study the host-pathogen interaction in actinomycetoma by *N. brasiliensis*, we have developed a murine experimental model [14]. Innate, Th1 and Th2 type immunity are involved in the host response against *N. brasiliensis* [19], but *N. brasiliensis* is able to escape from microbicidal mechanisms and multiply both extracellularly and within macrophages [20]. We focused our attention to define the lineage of foamy cells in actinomycetoma.

It is generally accepted that macrophages are the precursors of foamy cells [4], [10], but there are indications that they might arise from dendritic cells (DC) [21], [22]. Lineage studies of foamy cells are mainly based on surface markers, which are difficult to interpret as the surface markers are not exclusive to either cell type and/or markers may be acquired or lost depending on life cycle or activation status. In order to be conclusive about whether macrophages, DC or both are the precursors of foamy cells, we carried out *in vitro* and *in vivo* studies. We present strong evidence that not only macrophages but also dendritic cells become foamy cells in either *in vitro* infection or an *in vivo* experimental actinomycetoma model by *Nocardia brasiliensis*.

## Materials and Methods

### Mice

BALB/c mice were cared for and handled according to the International Review Board regulations and the Mexican Animal Protection Law (NOM-062-ZOO-1999), and were given Purina rodent food and distilled water *ad libitum*. The Bioethics Committee of the Facultad de Medicina at the Universidad Autónoma de Nuevo León approved this study under registration IN 10-003.

### Generation of Bone Marrow-derived Macrophages (BMDM) and Dendritic Cells (BMDC)

Mice were anesthetized and sacrificed by cervical dislocation. Bone marrow cells of femurs and tibiae of donor BALB/c mice were collected, treated with ammonium chloride [23] and washed in complete RPMI (RPMI-1640/25 mM HEPES/24 mM bicarbonate supplemented with 50–100 U penicillin/mL, 50–100 µg streptomycin/mL, (all Sigma-Aldrich, St.Louis, MO, USA; RPMI) and 10% heat-inactivated fetal bovine serum (Mediatech, Manassas, VA, USA) or complete advanced DMEM (advanced DMEM medium supplemented with 25 mM HEPES, 50–100 U penicillin/mL, 50–100 µg streptomycin/mL, (all Sigma-Aldrich, St.Louis, MO, USA) and 5% heat-inactivated fetal bovine serum (Mediatech, Manassas, VA, USA). Cells were plated at 1×10^5^ cells/cm^2^ in complete RPMI or complete advanced DMEM with their respective differentiation factors: 1) 30% L929-conditioned medium for BMDM [24], and 2) 20 ng/mL recombinant murine granulocyte-macrophage colony stimulating factor (GM-CSF) and 20 ng/mL interleukin 4 (IL-4) (Peprotech, Rocky Hill, NJ, USA) for BMDC. Media were refreshed every 2–3 days and cells were allowed to differentiate during 6 days. As negative control, we used non adherent cells from mouse spleen. In order to obtain these cells, spleen was removed under sterile conditions to prepare a cell suspension. Mononuclear cells were isolated using a ficoll diatrizoate density gradient and plated at 1×10^5^ cells/cm^2^, incubated in complete advanced DMEM overnight. Non-adherent cells were obtained and used in adoptive transference assays.

### Labeling of Cells

Cells were labeled with 2 µM carboxy-fluorescein diacetate succinimidyl-ester (CFSE; Molecular Probes/Invitrogen, Eugene, OR, USA) according to the manufacturer’s instructions. Viable cells that had been harvested with the use of scrapers one day after CFSE-labeling were counted by trypan blue exclusion using a hemocytometer. In the case of BMDC, ‘contaminating’ neutrophils were eliminated by washing the adherent cell layer 2–3 times with 10 mM phosphate buffered saline, pH 7.3 (PBS), whereas macrophages were depleted with the aid of streptavidin-conjugated magnetic beads (Miltenyi Biotec, Auburn, CA, USA) according to the manufacturer’s instructions. Briefly, the cell suspension was concentrated in 100 µL and incubated with biotin-conjugated anti-F4/80 (clone BM8, e-Biosciences, San Diego, CA, USA) diluted 1∶100 in complete RPMI for 15 min at room temperature plus 30 min at 4°C. After washing, the cell suspension was incubated with the magnetic beads, washed and passed through an MS-column (Miltenyi).

### Immunophenotyping of Surface Markers by Flow Cytometry

To block Fc-receptors, cells, 5×10^5^/50 µL complete RPMI, were incubated with murine hyperimmune serum (generated against *N. brasiliensis*) diluted 1∶50 for 15 min. at room temperature. Then, 1 volume of 2x solutions of specific conjugated antibody mixtures in complete RPMI were added: 1) anti-CD11b-allophycocyanin (1∶150, M1/70)+anti-CD11c-phycoerythrin (1∶40, N418), 2) anti-F4/80-phycoerythrin (1∶80, BM8)+anti-CD205-allophycocyanin (1∶625, 205 yekta), all e-Biosciences, and 3) the various phycoerythrin-conjugated isotype controls were anti-rat IgG2a, anti-rat IgG2b, and anti-hamster IgG (1∶50, all Caltag/Invitrogen, Carlsbad, CA, USA). After incubation in the dark for 15 min. at room temperature plus 30 min. at 4°C, the cells were washed 3 times with 2 mL PBS (400×g, 8 min.) and suspended in 550 µL 1% formalin/PBS or fixed in 10% formalin/PBS (30 min 4°C) and washed again 3 times in PBS to be suspended in 500 µL PBS. At least 1×10^4^ cells were analyzed using a FACSCalibur or FACS Canto II cytometer (BD Biosciences, Mountain View, CA) and Pro Quest or FACS Diva software (BD Biosciences, San Jose, CA).

### Phagocytosis Assay

1×10^6 ^BMDM or BMDC cells suspended in complete RPMI were seeded and allowed to adhere for 2 h in normal culture conditions before being incubated with a 30-fold excess of 2 µm Latex beads (Polysciences, Warrington, PA, USA) for 3 h at 37°C. After extensive washing with PBS, cells were fixed in 10% formalin and 100 cells were counted (in duplicate) and the percentage of phagocytosis was determined.

### *In vitro* Infection of BMDM and BMDC

BMDM and BMDC were seeded in 8-well Permanox slide chambers (Lab-Tex Thermo Fisher Scientific, Rochester, NY) at 5×10^4^ cells per well in 700 µL of advanced DMEM medium/5% heat-inactivated fetal bovine serum (Mediatech, Manassas, VA, USA) without antibiotics, and incubated at 37°C in 5% CO_2_ for 2 h. BMDM and BMDC were infected for 2 h with log-fase *N. brasiliensis* strain (ATCC no. 700358), cultured and recovered as described in [16], at a multiplicity of infection of 5∶1, After the removal of extracellular bacteria, cells were incubated for an additional 48 h in advanced DMEM/5% heat-inactivated fetal bovine serum without antibiotics before lipid body staining.

### Lipid Body Staining

Monolayers were washed 3 times with a sterile 0.85% saline solution and fixed in 10% formalin for 10 min. After the addition of 60% isopropanol, the chamber was removed and BMDM and BMDC were stained with Oil Red O (Sigma-Aldrich) for 15 min. Slides were rinsed with 60% isopropanol and counterstained with hematoxylin.

### Adoptive Transfer and Cell Tracing in the Actinomycetoma

Female BALB/c mice (8–10 wk) were infected with 10^6^ colony-forming units of *N. brasiliensis* into the left rear footpad as previously described [14]. At day 15 or 30 of the infection, 2×10^4^–5×10^5^ CFSE-labeled DC, macrophages or non-adherent control cells/100 µL PBS were injected into the lesions or the left rear footpad of control mice; 7 days later, biopsies of lesions were fixed in 10% formalin/PBS for 8–24 h, washed 3 times in PBS and saturated with 30% sucrose/PBS before being included in OCT to cut 10-µm cryostate sections. For detection of lipid droplets, slides were stained with Nile Red (Molecular Probes; 300 ng/mL in PBS from a 1 mg/mL stock in methanol). After washing the slides in PBS, they were mounted with Vectashield including 4′,6-diamidino-2-phenylindole (DAPI) (Vector Laboratories, Burlingame, CA, USA). Throughout the process, exposure to light was kept to a minimum. Slides were observed with a confocal laser scanning microscope (LSM700 or LSM710; Zeiss or TCS SPS; Leica) using a 20x/0.8 M27 Plan-Apochromat objective and a 30–39 µm pinhole with the following excitation/emission wavelength (λ_ex/em_) settings: λ_ex/em_ 360/458–531 for DAPI, λ_ex/em_ 488/490–569 for CFSE; and λ_ex/em_ 591/594–692 nm for Nile Red. Image analysis was performed with ZEN2009 or LAS AS software.

### Statistics

Student’s T test was used to detect significant differences between CFSE-labeled cells and control cells. A *P*-value <0.05 was considered significant.

## Results and Discussion

**Figure 1:**
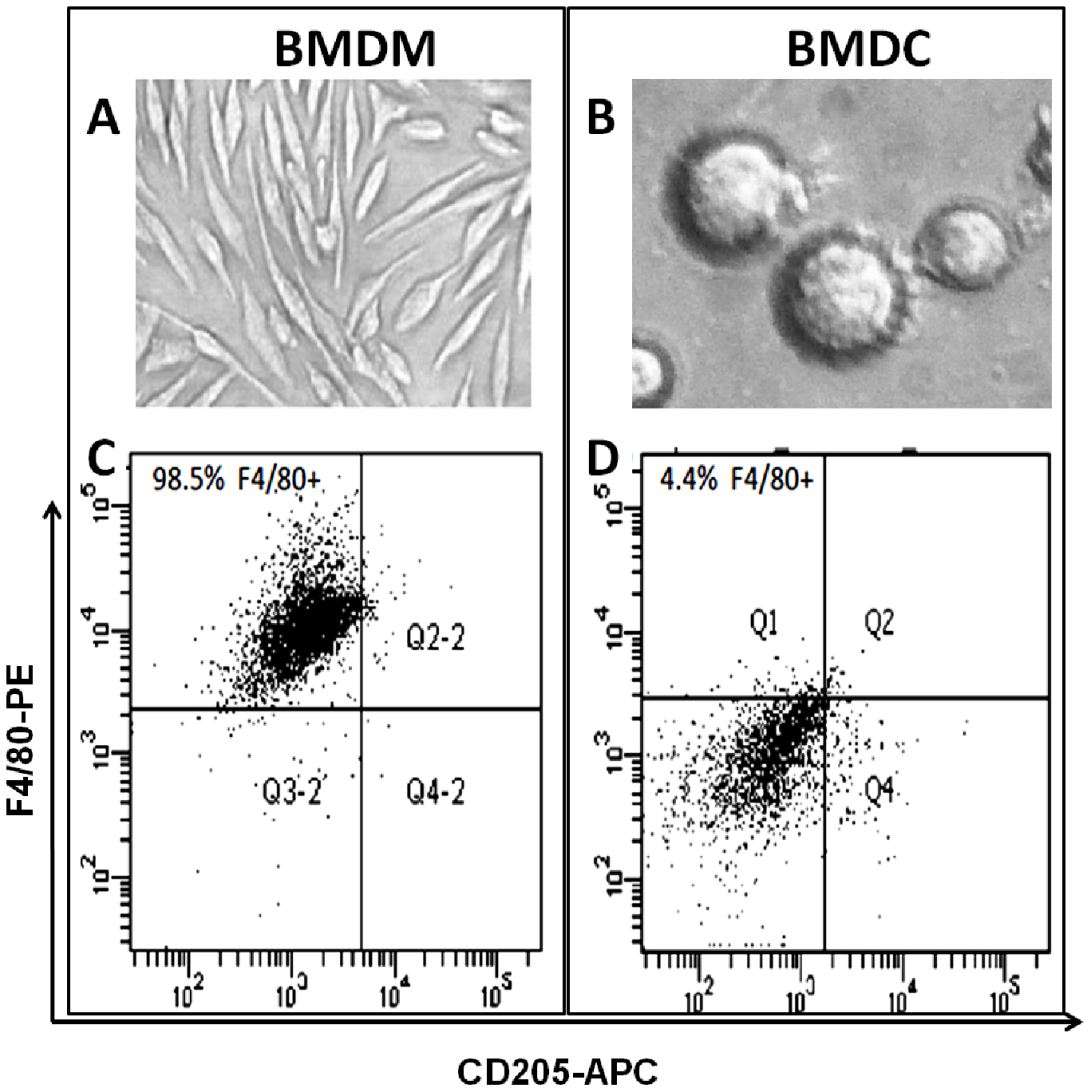
Morphology and immunophenotype of bone marrow-derived macrophages (BMDM) and dendritic cells (BMDC). Five-day cultures of BMDM generated with 30% L929-conditioned medium (A) or BMDC generated with 20 ng/mL GM-CSF and IL-4 (B); images were taken at 200x+digital zoom. Over 98% of the BMDM were F4/80+ (C), but less than 4.5% of the macrophage-depleted BMDC were F4/80+ (D).

Murine BMDM are easily generated *in vitro* with L929-conditioned medium as can be verified by their homogeneous morphology (fig. 1 a) and F4/80 expression in over 98% of the harvested cells (fig. 1c). On the other hand, murine bone marrow cells differentiated with GM-CSF and IL-4 generated a mixture of semi-adherent DC, adherent macrophages, and neutrophils in suspension [25]. The latter were easily removed by elimination of the medium and washing of the adherent cells. However, considering the aim of the study, it was most important to deplete all macrophages from the DC cell cultures. We did not rely on the commonly applied technique of positive selection of CD11c-labeled or CD205-labeled cells because macrophages tend to be CD11c+ [26], [27], and CD205+ macrophages have been described [21], [28]. Therefore, we chose for depletion of F4/80+ cells. F4/80 is considered a macrophage marker, although a subpopulation of DC, for example epidermal Langerhans cells and kidney DC, might also be F4/80+ [29], [30], [31]. To ensure DC (fig. 1b), we preferred losing some DC above having a macrophage contamination. Figure 1d demonstrates that less than 5% of the macrophage-depleted BMDC were F4/80+.

**Figure 2:**
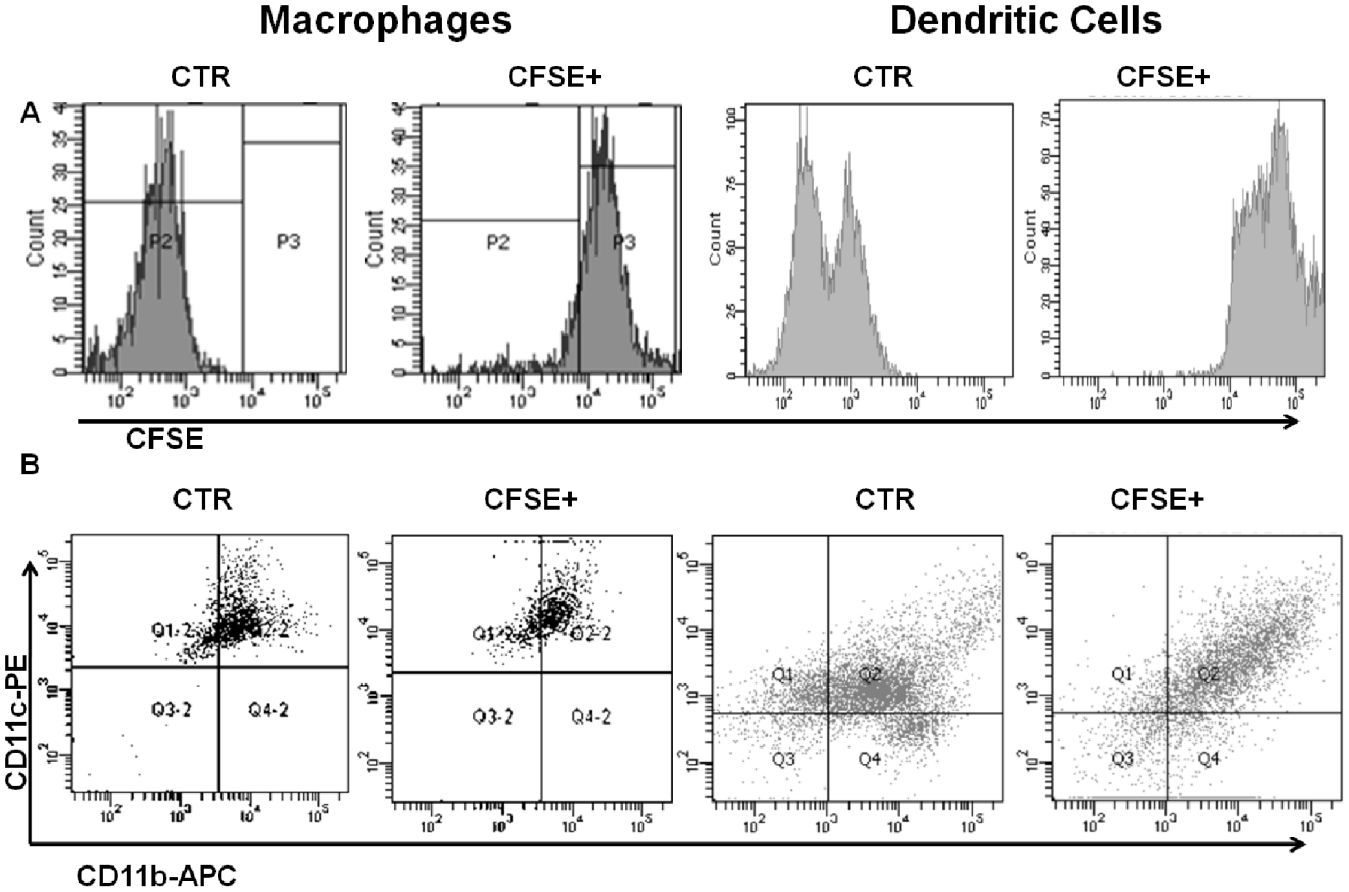
CFSE-labeling does not alter phenotype. A) BMDM and BMDC were fluorescent-labeled with 2 µM CFSE. B) Representative dot plots (1 out of 3 independent experiments) show that control (CTR) and CFSE-labeled macrophages (CFSE) had similar CD11b and CD11c expression profiles.

**Figure 3:**
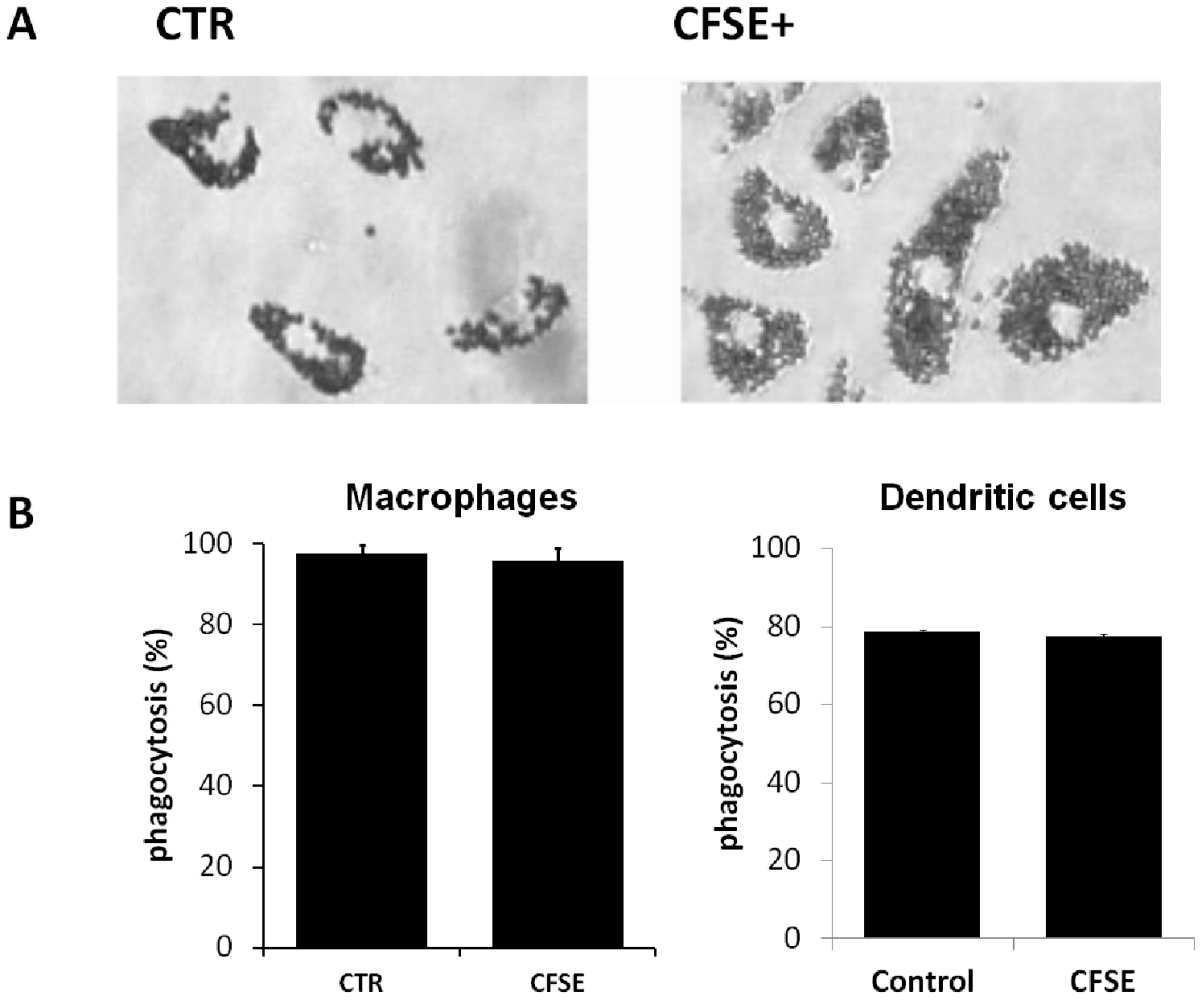
CF.SE-labeling does not alter functionality. The phagocytosis capacity of control and CFSE-labeled BMDM or CFSE-labeled BMDC were similar; 100 cells were counted; the experiment was repeated 3 times; *P*>0.05.

Although CFSE labeling (fig. 2a) affected BMDM and BMDC morphology at the short term with cells rounding up during the first hours after labeling, the next day, viable cells had recovered typical morphology and adherence. The immunophenotype pattern was hardly altered between control and CFSE-labeled BMDM or BMDC (fig. 2b) and considered acceptable. Similarly, functionality was unaffected as verified by phagocytosis studies of harvested and reseeded CFSE-labeled and control BMDM, which had phagocytosis indices of 96.0% (±2.96) and 97.6% (±2.14), respectively; meanwhile CFSE-labeled and control BMDC had phagocytosis indices of 78.6% (±4.1) and 77.6% (±5.8), respectively (fig. 3).

**Figure 4:**
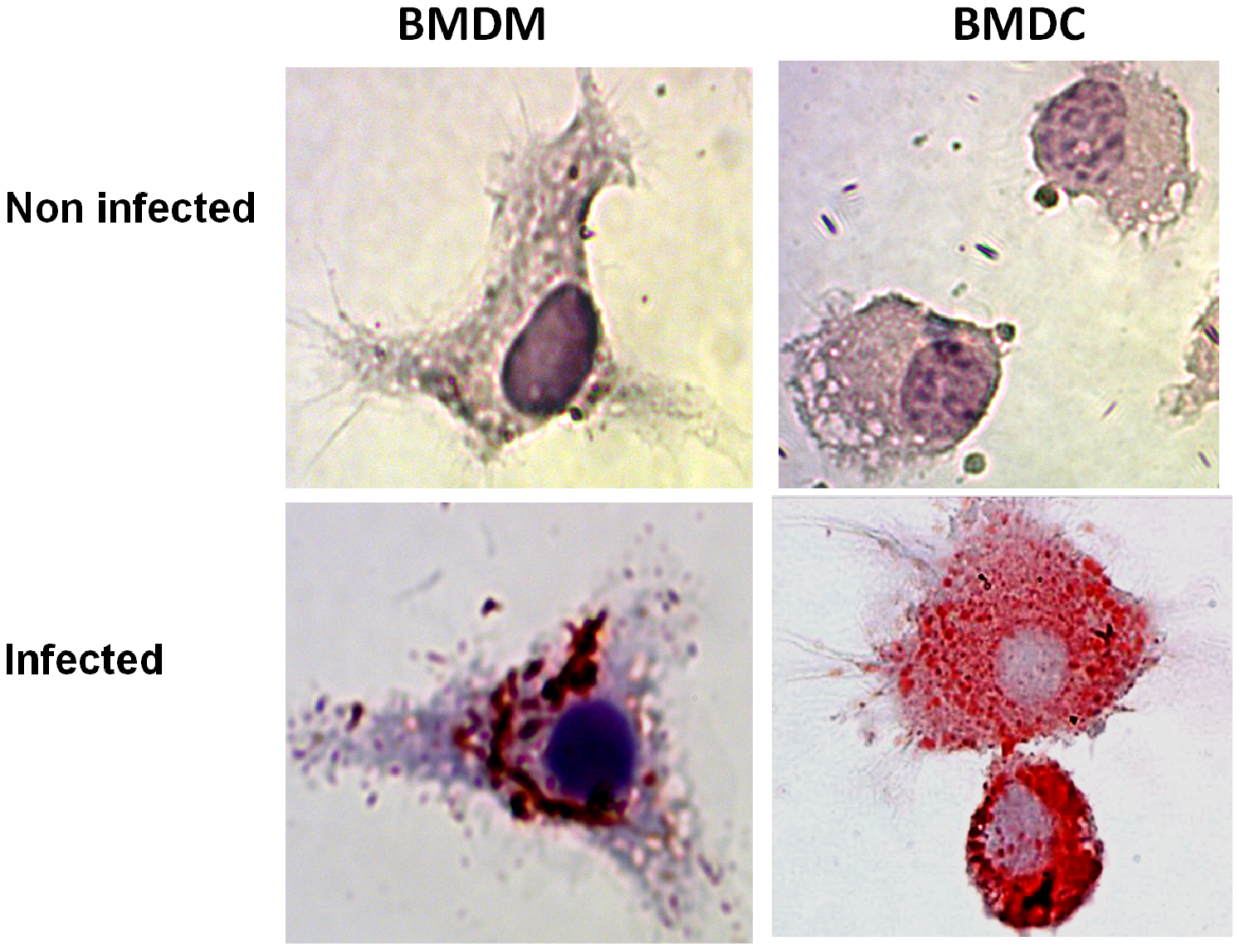
Bone marrow-derived macrophages (BMDM) and dendritic cells (BMDC) become lipid-laden after *in vitro* infection with *N. brasiliensis*. Non-infected BMDM and BMDC (A) and BMDM and BMDC infected with *N. brasiliensis* were stained with Oil Red O as described in Materials and Methods. Only infected macrophages and dendritic cells were Oil Red O positive. (Images were taken at 100×magnification).

**Figure 5:**
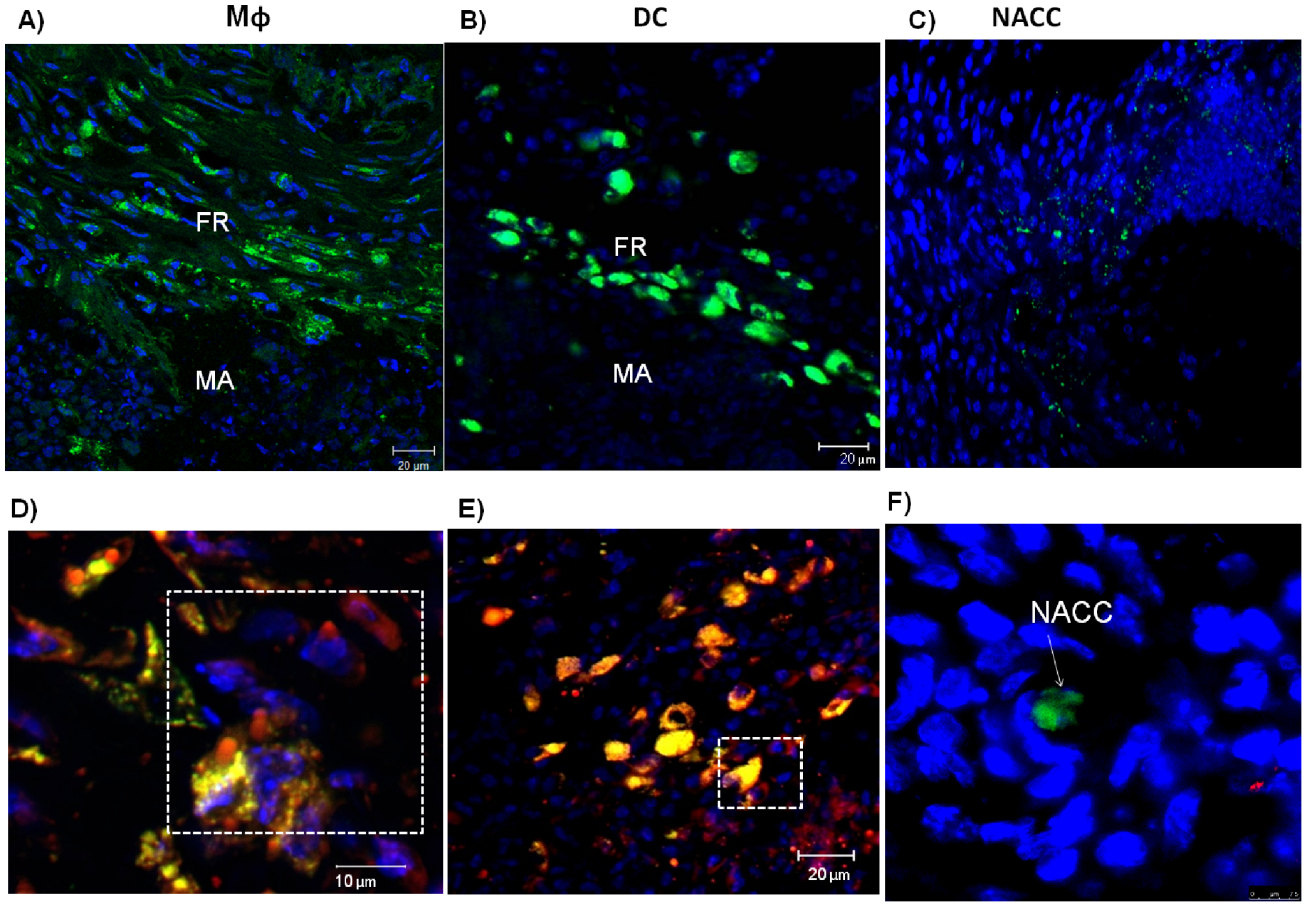
Transferred CFSE-labeled macrophages (Mφ) and dendritic cells (DC) become lipid-laden. Macrophages (Mφ; in A) and dendritic cells (DC; in B) transferred at day 30 of a *N. brasiliensis* infection are 7 days later localized in the fibrotic ring (FR) of a microabscess (MA). Non-adherent spleen control cells (NACC; in C) transferred at day 30 of a *N. brasiliensis* infection are 7 days later localized outside of the FR and MA. Nile Red staining of lipid droplets is observed in transferred Mφ (D) and DC (E). Transferred cells are stained green by CFSE, lipid droplets are stained red by Nile Red, and nuclei are stained blue with DAPI.

The *in vitro* study revealed that BMDM and BMDC became foamy cells as both accumulated lipids into their cytoplasm after infection with *N. brasiliensis,* whereas uninfected BMDM and BMDC did not (fig. 4). CFSE-labeled BMDM and BMDC, transferred into 30-days lesions of experimental actinomycetoma induced by *N. brasiliensis,* could be traced 7 days later interspersed into the fibrotic ring of multilocular microabscesses, the typical site of foamy cells. The lipid droplet fluorophore Nile Red co-localized within the transferred cells, either BMDM or BMDC, as well as in recipient’s own cells that had accumulated lipid droplets (fig. 5). On the other hand, non-adherent control cells were localized out of fibrotic area and did not accumulate lipid droplets. Thus, we demonstrated that macrophages *and* DC can be differentiated into foamy cells, both *in vitro* and *in vivo.*

The ability of both cell types to become lipid-laden further diminishes the supposed difference between them. Although separate lineages for either cell type have been reported [32], both 1) can differentiate from monocytes [26], 2) share many cell surface markers in fact, it is hard to find an exclusive surface marker, 3) can present antigens, and 4) can be classically activated, known as M1 macrophages or TipDC, respectively [33], [34]. Moreover, recently our group reported that *N. brasiliensis* modulates the local immune system to favor chronic disease and bacterial. The local microenvironment is a characterized by the expression of inflammatory (interferon-gamma) and anti-inflammatory (IL-10 and IL-13) cytokines [35]–[36]. This immunological environment may play an important role in foamy cell formation and bacterial survival. The mechanism for lipid droplet accumulation in *N. brasiliensis* infected macrophages and DC remains to be elucidated, however the findings from infections with some mycobacterial species may apply because they share a similar cell wall composition and genetic background. The accumulation of cholesterol and cholesterol esters in macrophages infected with *Mycobacterium leprae* [37] may favor intracellular survival in at least two ways as it facilitates mycobacterial entry and is involved in the inhibition of phagosome-lysosome fusion [38]. The involvement of Toll-like receptors, especially TLR-2 and TLR-6 has been studied in *Mycobacterium leprae* pathogenesis [10] [39]. As a consequence of the activation of the innate immune response, membrane lipids are liberated to generate eicosanoid immunomodulators, such as prostaglandin E (PGE), which accumulate inside the cell [7], [10]. Oxygenated mycolic acids, or the oxygenated lipids generated because of reactive oxygen species, induce the expression of lipid scavenger receptors and thus facilitate lipid uptake and the formation of lipid-laden cells [4] [40]. Key regulators of lipid metabolism are a family of lipid sensor nuclear receptors, which include three types of peroxisome proliferator-activated receptors (PPAR), PPARα, PPARβ/δ, and PPARγ [41]. A variety of endogenous lipids and synthetic ligands stimulate PPAR to induce gene expression that finally results in lowering circulating lipid levels, In addition, PPAR agonists have significant anti-inflammatory activities [41], [42], which may be independent of PPAR [43], These PPAR, especially PPARγ, have received most attention in studying molecular mechanisms of lipid accumulation in mycobacterial infected cells [44], [45]. Virulent, but not avirulent, mycobacterial infection induced a TLR-2 dependent PPARγ expression that correlated with lipid droplet accumulation [44], which in turn correlated with intracellular pathogen survival [45]. Furthermore, PPARγ modulated the cytokine profile of macrophages infected with the attenuated *M. tuberculosis* strain H37Ra by diminishing pro-inflammatory signaling and favoring the anti-inflammatory cytokine IL-10 [45]. The anti-inflammatory cytokine profile favors alternative activation of macrophages and pathogen survival. We previously reported the local induction of an anti-inflammatory environment with increased expression of IL-13 and IL-10 in our actinomycetoma model [35], [36], Any of these mechanisms may be involved in accumulation of lipids in either macrophages or DC in the *N. brasiliensis* infection, that may be studied in the future.

In conclusion, we present strong experimental evidence, that macrophages and DC differentiate to foamy cells in vitro and in vivo infections.
